# Progress in Research of Nanotherapeutics for Overcoming Multidrug Resistance in Cancer

**DOI:** 10.3390/ijms25189973

**Published:** 2024-09-16

**Authors:** Ayitila Maimaitijiang, Dongze He, Dingyang Li, Wenfang Li, Zhengding Su, Zhongxiong Fan, Jinyao Li

**Affiliations:** School of Pharmaceutical Science (Institute of Materia Medica) & College of Life Science and Technology, Xinjiang University, Urumqi 830017, China

**Keywords:** nanotherapeutics, multidrug resistance cancer, molecular mechanisms, therapeutic strategies

## Abstract

Chemotherapy has been widely applied in oncotherapy. However, the development of multidrug resistance (MDR) has diminished the effectiveness of anticancer drugs against tumor cells. Such resistance often results in tumor recurrence, metastasis, and patient death. Fortunately, nanoparticle-based drug delivery systems provide a promising strategy by codelivery of multiple drugs and MDR reversal agents and the skillful, flexible, smart modification of drug targets. Such systems have demonstrated the ability to bypass the ABC transporter biological efflux mechanisms due to drug resistance. Hence, how to deliver drugs and exert potential antitumor effects have been successfully explored, applied, and developed. Furthermore, to overcome multidrug resistance, nanoparticle-based systems have been developed due to their good therapeutic effect, low side effects, and high tumor metastasis inhibition. In view of this, we systematically discuss the molecular mechanisms and therapeutic strategies of MDR from nanotherapeutics. Finally, we summarize intriguing ideas and future trends for further research in overcoming MDR.

## 1. Introduction

Following the identification of oncogenes and tumor suppressor genes in the 1980s, researchers have gained a deeper understanding of the molecular regulation of various tumors and treatment options involved in the initiation, development, and metastasis of tumors [1]. Upon the approval of imatinib by the Food and Drug Administration (FDA) in 2002, oncotherapy officially entered the era of targeted therapy [2]. Recently, the rapid development of chemotherapy drugs, targeted drugs, and immunotherapy has significantly improved cancer patients’ survival rate and life quality. However, these small-molecule drugs often encounter multidrug resistance (MDR) during therapy due to their different action mechanisms and structural characteristics. MDR in malignant tumors is one of the main reasons for cancer chemotherapy failure. Therefore, how to explore a widely effective treatment strategy to reverse multidrug resistance is an important research direction in oncotherapy.

MDR, initially proposed by Biedler and Riehm in 1970, is a multifactor-mediated phenomenon that involves varying levels of therapeutic resistance in tumor cells towards chemotherapeutic drugs with distinct structures and targets [3,4]. Once resistance to anticancer drugs develops, cancer cells can rapidly metastasize, ultimately leading to patient mortality. MDR poses the most significant challenge in cancer chemotherapy. Therefore, it is critical to overcome multidrug resistance in various tumors and restore the sensitivity of cancer cells to chemotherapeutic agents, targeted drugs, and immunotherapy [5,6]. MDR is typically categorized into two main types: intrinsic resistance, which relates to the ability of cancer cells to evade treatment due to their inherent factors, and acquired resistance, which describes the gradual development of anticancer drug resistance during treatment. Conquering both types of resistance necessitates a comprehensive understanding of potential targets and multiple mechanisms of drug resistance [7].

Nanoparticles (NPs) are rapidly advancing in cancer therapy, surpassing the limitations of conventional small-molecule chemotherapy drugs. Nanotherapy has emerged as an innovative and promising approach to chemotherapy, aiming to effectively target tumors and overcome multidrug resistance (MDR). This novel and multifunctional type of NPs is highly favored by researchers due to its capability of carrying two or more different dosage forms or sizes of drugs simultaneously. This capacity enhances the efficacy of chemotherapy while reducing its side effects. Several studies have focused on designing nano-codelivery systems that can transport traditional chemotherapy drugs and multiple MDR transporter inhibitors. These systems function by interfering with the “efflux pump” functions of cell membrane transporters, ultimately increasing intracellular drug concentrations to combat tumor MDR.

## 2. Mechanisms of MDR

**Figure 1:**
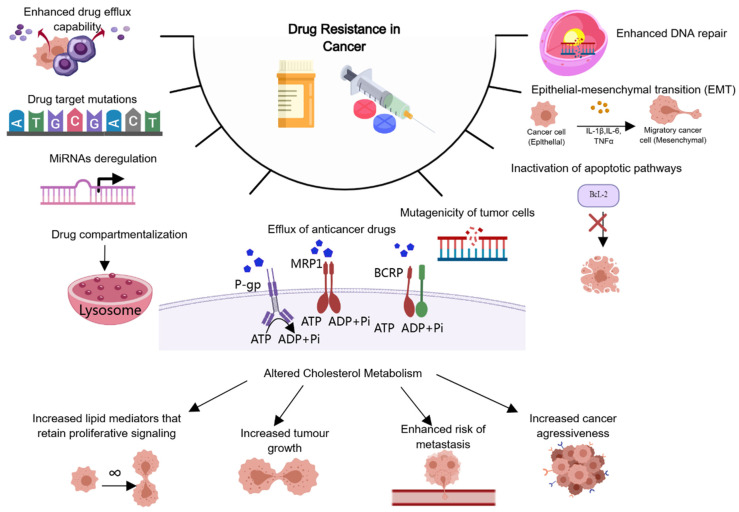
Mechanisms of anticancer drug resistance: efflux pump-mediated mechanisms of MDR and efflux pump-independent drug resistance mechanisms.

The emergence of MDR in cancer is typically the result of multiple mechanisms. The activation of drug efflux pumps is the final step in MDR. These mechanisms include ATP-dependent drug efflux, inhibition of the apoptosis pathway, DNA repair, and tumor tissue heterogeneity (Figure 1). These mechanisms can occur independently or in combination, reducing the toxicity of drugs to tumor cells. MDR affects most classical anticancer drugs, such as anthracyclines (doxorubicin and daunorubicin), vinca alkaloids (vinblastine and vincristine), RNA transcription inhibitor actinomycin D, and microtubule stabilizing drug paclitaxel, among others [7,8].

### 2.1. ATP Transporter Cassette (ABC Transporter Protein) Superfamily

**Figure 2:**
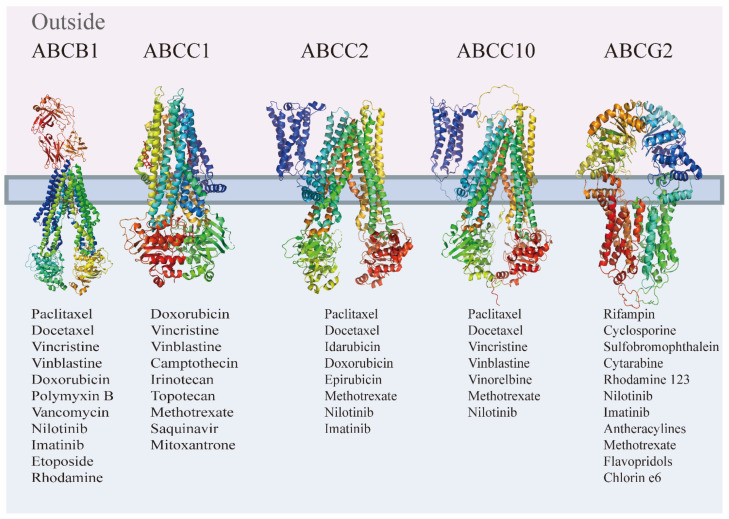
ABC transporter superfamily partial members, and their substrates.

The primary reason for MDR is the overproduction of ABC transporters. These transporters are fundamentally composed of two distinct conserved domains: the ATP-binding domain that is upregulated, also referred to as nucleotide-binding domains (NBDs), and the transmembrane domain (TMD) [9]. Currently, there are 48 human ABC genes categorized into seven subfamilies: ABCA, ABCB, ABCC, ABCD, ABCE, ABCF, and ABCG, based on their homologous sequences and major structures [10]. Under normal physiological conditions, ABC proteins are widely expressed in human organs and primarily responsible for substrate transport. However, in resistant cells, these transporter proteins are highly expressed, resulting in the exclusion of chemotherapy drugs from cancer cells, reduction in intracellular drug concentration, and isolation of target drugs outside of the cell membrane. This process makes cancer cells multidrug-resistant. The ABC transporter superfamily comprises numerous members, and Figure 2 summarizes some of these family members and their respective substrates [11,12,13,14,15]. P-glycoprotein 1 (P-gp/ABCB1), multidrug resistance-associated protein (MRP/ABCC1), and breast cancer resistance protein (BCRP/ABCG2) are among the main reasons for chemotherapy resistance.

#### 2.1.1. P-gp

ABCB1 (P-gp) is a membrane protein encoded by the MDR1 gene and was initially discovered by Ling and colleagues in 1976 [16]. It has a molecular weight of 170 KD. P-gp is an ATP-binding cassette (ABC) transporter protein with significant physiological functions. Through utilizing ATP hydrolysis, the nucleotide domains (NBDs) of P-gp undergo conformational changes in the transmembrane domains (TMDs), enabling an efficient efflux of anticancer drugs from the cell [5,17]. This efflux mechanism primarily involves the expulsion of large amounts of amphiphilic and non-ionic substances, such as paclitaxel and anthracyclines. The conformational changes in P-gp play a crucial role in the development of MDR in cancer cells and the reduction in the effectiveness of anticancer drugs. On the other hand, there has been extensive research on P-gp-targeted inhibitors, including competitive inhibitors like cyclosporine A [18] and non-competitive inhibitors like verapamil [19]. These inhibitors interfere with P-gp-mediated transport of small molecules, ultimately leading to enhanced intracellular accumulation of chemotherapy drugs.

#### 2.1.2. MRP1

MRP1 was first found in resistant cell lines and is a 190 KD protein on the cell membrane. Unlike P-gp, human MRPs can transport organic anionic sulfates, glucuronides, and other substances while also excreting various exogenous or endogenous substances outside the cell [20,21]. The unique structure of MRPs results in different drug transport and accumulation patterns compared to P-gp. The substrate of MRP1 can be directly recruited from the cytoplasm, whereas P-gp selectively binds substrates from the lipid bilayer structure [22]. Recent studies have indicated that the activity of glutathione peroxidase can be increased by the GSH synthase inhibitor Buthionine Sulfoximine (BSO) in human embryonic kidney cells, which further regulates MRP1-dependent vincristine resistance [23]. These findings indicate that MRP1-mediated MDR may be achieved by promoting the flow of glutathione-binding drugs from cancer cells.

#### 2.1.3. BCRP

BCRP (Breast Cancer Resistance Protein), a 72 kD semi-transporter protein composed of six transmembrane amino acid fragments [24], is initially detected in malignant breast cancer. Despite its structural differences from P-gp, BCRP shares a significant number of substrates with P-gp, such as anthracyclines, anthracene, daunorubicin, and doxorubicin hydrochloride. Studies have identified that arginine (Arg or R) is positioned at the carboxyl end of the third transmembrane segment of the transmembrane domain, potentially forming a salt bridge that enhances substrate binding and facilitates the efflux of drugs from the cell [13,25]. Certain inhibitors, including lapatinib [26], erlotinib, and gefitinib [27], have already been identified as effective in inhibiting BCRP-mediated resistance, with some of these drugs having been validated in clinical trials.

#### 2.1.4. ABCG2 and Photosensitizer Resistance

Photodynamic therapy (PDT) is a treatment approach that requires the administration of a photosensitizer, usually a photosensitizing medication, into cells or tissues. The photosensitizer is then activated by light wavelengths, initiating a chemical reaction that leads to the elimination of the affected tissue. The photosensitizer sodium chloride e6 (Ce6) has been shown to accumulate inadequately in tumor cells, potentially diminishing the efficacy of tumor destruction. In glioblastoma multiforme (GBM) stem cells, the high expression of ABCG2 leads to decreased accumulation of Ce6, which may limit the effectiveness of PDT. The activity of ABCG2 can be inhibited by drugs such as KO143. A study demonstrated that short-term inhibition with these drugs significantly enhanced the sensitivity of GBM stem cells to PDT, with fewer side effects compared to chemotherapy [28]. In their study, the scientists explored the role of ABCG2 in regulating Ce6 accumulation and phototoxicity, employing two glioblastoma cell lines: U251-V and U251-EV. They observed that augmenting ABCG2 expression in U251-V cells caused a reduction in phototoxicity. However, this trend was reversed upon administering KO143 to inhibit ABCG2, resulting in a heightened phototoxicity that exceeded that seen in the non-induced control cells.

Additionally, PDT makes use of 5-aminolevulinic acid (5-ALA), a precursor of a selective photosensitizer, to synthesize the fluorescent protoporphyrin IX (PpIX), including phototoxic effects within cells [29]. When excited by visible light at 635 nm, PpIX generates reactive oxygen species, including singlet oxygen, which obliterate tumor tissues through their toxicity. This leads to tumor vascular obstruction, immune system activation, as well as apoptosis and necrosis of tumor cells. The overexpression of PpIX as a substrate of ABCG2 may result in diminished PDT efficacy. To investigate the impact of ABCG2 expression on the intracellular accumulation of PpIX, researchers noted that Dox-treated cells with ABCG2 overexpressing cells exhibited the lowest average fluorescence intensity of PpIX. Conversely, cells with an empty expression plasmid showed the highest PpIX concentration [29]. Furthermore, inhibiting ABCG2 using KO143 restored PpIX accumulation, thereby demonstrating a significant correlation between ABCG2 expression levels and intracellular PpIX content.

### 2.2. Induction of Anti-Apoptotic Mechanisms

The cytotoxicity of chemotherapy drugs largely depends on the cell cycle of cancer cells induced by drugs, which further affects the ability of cell apoptosis. Platinum-based chemotherapy drugs, such as cisplatin, are alkylating agents that bind to DNA and create intra- and inter-chain crosslinking, leading to DNA damage and ultimately triggering mitochondrial-mediated apoptosis. The apoptotic pathway is predominantly regulated by pro-apoptotic proteins from the BcL-2 family (e.g., Bim, Bid, Bad, Bik, Bax, Noxa, and Bmf), as well as anti-apoptotic proteins (e.g., BcL-2, BcL-w, Mcl-1, Bfl-1, and BcL-xL) [30,31]. Blocking pro-apoptotic proteins or boosting anti-apoptotic proteins can make tumor cells more resistant to chemotherapy and stop them from dying [32].

### 2.3. Changes in Intracellular Enzyme Activity

Compared to normal cancer cells, there are changes in the enzyme activities of protein kinase C (PKC), topoisomerase II (Topo II), and glutathione-S-transferase—π (GST—π) in multidrug-resistant cell lines. PKC can speed up phosphorylation of P-gp, resulting in MDR [33]. Decreased Topo II activity can contribute to drug resistance in tumor cells. Studies have shown that inhibitors of topoisomerase I, including irinotecan and topotecan, as well as inhibitors of topoisomerase II, such as etoposide, teniposide, and anthracyclines like idarubicin, daunorubicin, and doxorubicin, interfere with the activity of these enzymes during DNA replication, resulting in DNA fragmentation [34]. Combining GST—π with lipophilic anticancer drugs can enhance their water solubility, facilitate drug efflux, and thus weaken the sensitivity of anticancer drugs towards tumor cells.

### 2.4. ROS Promotes the Occurrence and Development of MDR

Numerous studies have demonstrated the involvement of reactive oxygen species (ROS) in and their regulation of various cellular physiological processes. These processes include apoptosis, gene expression, gene mutations, and activation or inhibition of cell signaling pathways [35]. ROS are typically induced by cellular metabolism or external factors, and they play a crucial role in regulating oxidation-reduction responses, oxidative stress, and related signaling pathways in cancer development. Examples of these pathways include the oxidative stress MAPK signaling pathway, PI3K/AKT signaling pathway, and NF-κB signaling pathway [36]. ROS activation leads to oxidative stress and cellular damage, ultimately resulting in cell death through oxidative stress mechanisms. In this process, ROS acts as an activator of oxidative stress, which induces apoptosis and death. However, most cancer cells can survive under oxidative stress activation by evading apoptosis pathways and developing resistance to chemotherapy drugs. Research suggests that cancer cells exhibit higher ROS release, a unique biochemical characteristic that could be exploited in cancer therapy. Attenuating defense mechanisms can induce cell apoptosis, as it reduces ROS levels. Drug-resistant cells that possess excessive antioxidant enzymes may exist in a higher oxidative stress environment. Eliminating enzymes involved in antioxidant defense can stimulate higher antioxidant stress, eventually leading to cell death. Therefore, ROS modulators could potentially play a critical role in overcoming multidrug resistance [37].

### 2.5. Induction of microRNA Regulatory Disorders

MiRNAs are a growing family of short noncoding RNAs that are frequently dysregulated in malignant tumors. They also have a crucial role in tumor drug resistance. Numerous studies have indicated that miRNAs are involved in the regulation of MDR in cancer, specifically in regulating drug resistance-associated proteins such as miR-21, miR-27, and miR-129, which inhibit P-gp expression. Additionally, miRNAs impact cell apoptosis by regulating BcL-2 through miR-497, miR-181b, and miR-204, and accelerate the process of epithelial–mesenchymal transition (EMT) through miR-1274a [38,39,40].

### 2.6. Enhancement of DNA Damage Repair Ability

DNA damage repair (DDR) is an important mechanism contributing to drug resistance in cancer cells. These cells can counteract drug-induced DNA damage and apoptosis through efficient repair processes [41]. The DNA repair endonuclease XPF and the DNA excision repair protein ERCC1, both involved in the nucleotide excision repair (NER) pathway, play crucial roles in repairing the DNA damage caused by crosslinking agents and platinum-based drugs. Increased expression of XPF and ERCC1 proteins has been found to be associated with the development of cisplatin resistance in cancer cells [42]. Furthermore, certain innovative compounds, including ERCCI-XDF inhibitors, have demonstrated potential in reestablishing the sensitivity of melanoma cells to cisplatin [43]. Furthermore, the process of mutagenic translation synthesis (TLS) in DNA damaged by chemotherapy is closely linked to the occurrence of MDR in cancer cells. Wojtaszak et al. demonstrated that JH-RE-06, a highly specific small-molecule inhibitor, effectively blocks the recruitment of the mutagenic polymerase POL involved in TLS activity. They also found that JH-RE, in combination with cisplatin, enhances cisplatin-induced cytotoxicity in mouse and human cancer cell lines [44].

### 2.7. Chemotherapeutic-Induced Epidermal–Mesenchymal Transformation

Chemotherapy drugs can induce epithelial–mesenchymal transition (EMT) in tumor cells, causing them to transform from epidermal to stromal types [45]. Tumor cells that undergo EMT gain stronger migration and invasion abilities, enabling them to easily migrate out of the primary lesion, invade blood vessels, enter the circulatory system, and ultimately establish a metastatic lesion in a distant organ [46]. Cancer cell EMT activation leads to increased invasiveness, metastatic potential, and drug resistance. A few transcription factors regulate EMT. These are mainly from the SNAIL, TWIST, and ZEB families [47,48].

Furthermore, alterations in drug targets, the existence of cancer stem cells, variations in the cellular microenvironment, excessive angiogenesis signaling, and genetic mutations arising throughout cancer progression all contribute to the emergence of cell types that can selectively endure multiple rounds of chemotherapy. The selective pressure exerted by the active elimination of drug-sensitive cancer cells may fuel the evolution of MDR [49]. Among them, the ABC (ATP-binding cassette family) transporters are a class of macromolecular membrane proteins. They rely on ATP energy to participate in a range of processes within normal cells, including the maintenance of osmotic pressure, nutrient uptake, and drug transport [50,51], However, ABC transporters are overexpressed in drug-resistant tumor cells, and the structure of these proteins dictates that they can recognize many types of substrates. Several previous studies have found that chemotherapy drugs induce the upregulation of drug resistance genes through a complex signal pathway, resulting in an increase in multidrug resistance proteins on tumor cell membranes, leading to MDR [52,53].

#### Cellular Plasticity

The phenotypic plasticity of cancer cells facilitates their dynamic response to changes in their surrounding environment, such as variations in oxygen levels, nutrient availability, and immune system stress [54]. This adaptability allows them to thrive in diverse environments and evade treatments. By altering their phenotype, cancer cells can resist various treatments, including chemotherapy, targeted therapy, and immunotherapy.

Epithelial–mesenchymal transition (EMT) is a process by which epithelial cells acquire mesenchymal characteristics, enhancing their migratory and invasive capabilities. Besides EMT, the phenotypic plasticity of cancer cells includes other transformation mechanisms, such as mesenchymal–epithelial transition (MET), which allows them to regain epithelial-like features, contributing to the development of new tumor areas or metastatic foci [55].

Cancer cells exhibit characteristics akin to stem cells, such as enhanced self-renewal and multidirectional differentiation potential [56]. These traits facilitate their adaptation to stress and enable them to resume growth after treatment.

### 2.8. The Tumor Microenvironment and Exosome-Mediated Drug Resistance

The impact of the tumor microenvironment (TME) on drug resistance is a significant research area within oncology. The term “tumor microenvironment” refers to the intricate milieu surrounding and within tumor tissues, encompassing not only tumor cells but also various other components such as stromal cells, the vascular system, immune cells, signaling molecules, the extracellular matrix (ECM), metabolites, etc. [57]. These components collectively influence tumor growth, progression, metastasis, and therapeutic response. Key factors affecting drug efficacy include tumor stroma, hypoxia, and diverse immune cells.

(1) The intricate tumor stroma, an intricate environment encircling the tumor, is comprised of non-tumoral cells, the ECM, blood vessels, and lymphatic vessels, all of these components engaging in mutual interactions with the tumor. The composition and structure of the tumor stroma significantly influence drug effectiveness, particularly through their impact on drug permeability. The ECM within the tumor stroma, which is typically dense and fibrous, often impedes drug penetration [58]. For instance, ECM components like collagen fibers and fibronectin can create a physical barrier that obstructs drugs from reaching tumor cells.
(2) The hypoxic state of tumor tissue is a critical component of the tumor microenvironment. Abnormal vascularization in tumors leads to a hypoxic milieu, which subsequently prompts metabolic changes in tumor cells, increasing their resistance to pharmacological treatments. Additionally, hypoxia can trigger the expression of hypoxia-inducible factors, HIF [59,60], allowing tumor cells to adjust to the hypoxic conditions by modulating the cell cycle, repair mechanisms, and metabolic pathways, thus enhancing their resistance to therapy. Moreover, hypoxia diminishes the effectiveness of chemotherapeutic agents. Specifically, it is the principal mechanism behind the resistance of tumor cells to photodynamic therapy (PDT) [61].
(3) The complex signaling and interactions among immune cells (e.g., macrophages, T cells, dendritic cells, etc.) within the tumor microenvironment play a pivotal role in drug resistance. Tumor-associated macrophages (TAMs), for example, are capable of secreting various factors (e.g., cytokines, chemotactic factors) that promote tumor cell survival and drug resistance [62]. Additionally, immune cells can enhance resistance by compromising the effectiveness of pharmaceutical agents or by activating on resistance-related pathways. Tumor cells may also avoid immune attack through several mechanisms. One aspect that is crucial is the manifestation of PD-L1, which disrupts the efficacy of T cells. This not only impacts the success of immunotherapy but also indirectly contributes to increased resistance to chemotherapeutic agents [63].

Exosomes are small membrane vesicles secreted by cells encompassing a diverse array of biomolecules, such as proteins, RNA, and microRNA. They play a vital role in intercellular communication. Within the scope of drug resistance in tumor cells, exosomes discharged by drug-resistant cells may promote resistance to chemotherapeutic agents by facilitating the transfer of proteins, RNA, and microRNA, as well as by altering the microenvironment [64].

(1) Exosomes possess the capacity to encapsulate and transfer drug resistance-associated proteins, such as transporter proteins and metabolizing enzymes, to sensitive cells. These proteins can modify drug metabolism and elimination in sensitive cells, thereby enhancing their resistance to drugs. The conveyance of ABC transporter proteins through exosomes serves as a mechanism to induce drug resistance. These proteins help drugs leave cells, reducing the number of drugs in the cells. P-gp from drug-resistant cancer cells can help get rid of drugs in other cells. Furthermore, the MCF-7/ADM human breast cancer cell is noted for expressing elevated levels of P-gp, and the high presence of P-gp in exosomes suggests the potential for the direct transfer of the P-gp protein via exosomes [65]. The internalization of exosomes containing P-gp results in the transfer of drug resistance traits to drug-sensitive cells.
(2) Additionally, exosomes facilitate the transport of specific RNAs and microRNAs (miRs), which modulate gene expression in recipient cells. Cancer stem cells possess the ability to metastasize and augment drug resistance in adjacent susceptible cancer cells by discharging exosome microRNA (miRNA) molecules targeting anti-apoptotic and immunosuppressive pathways. For instance, an article [66] indicates that exosome-mediated transfer of miR-21 from cancer-associated fibroblasts (CAFs) to ovarian cancer cells hinders cell death and fosters resistance to paclitaxel treatment by downregulating the expression of the apoptotic protease-activating factor (APAF1).
(3) Exosomes have been shown to facilitate drug resistance by releasing a variety of bioactive molecules that alter the tumor microenvironment [67]. For example, exosomes can modulate the function of immune cells, promote angiogenesis, or affect the tumor stroma, thereby providing further support for the survival and proliferation of drug-resistant cells [68].

## 3. Strategies to Overcome Multidrug Resistance

The molecular mechanisms elucidate various objectives that should be considered when addressing the development of MDR. In cancer drug therapy, the modification of drug targets, activation of downstream pathways, and inhibition of redundant parallel signaling cascades are crucial. Overcoming tumor drug resistance entails targeting oncogenic signaling pathways, such as Her2, EGFR, PI3K, AKT, mTOR, Ras, Raf, Mek, Wnt, and *β*-catenin. A different method consists of blocking the manifestation and operational capacity of ABC transporters, which augment drug expulsion and elicit MDR. Restoring the oxidative stress sensitivity of MDR cells to enhance the cytotoxicity of antitumor drugs, as well as identifying novel targets to restore cancer cell response to chemotherapies and immunotherapies, may serve as potential therapeutic strategies to overcome MDR [69]. The multifunctional nanoparticles highlighted in this article often attract researchers because of their ability to simultaneously carry two or more drugs of different polarities or different sizes. It was found that MDR can be overcome by designing a nano-codelivery system to simultaneously deliver one or two transporter-related inhibitors in addition to traditional chemotherapy drugs to interfere with the efflux of cell membrane transporters. Studies have shown that MDR can be surmounted by designing a nano-codelivery system that delivers one or two inhibitors targeting transporter proteins, in addition to traditional chemotherapy drugs, to obstruct drug efflux mediated by cell membrane transporters. This highlights the potential of nano-codelivery systems in overcoming tumor MDR.

### 3.1. Inhibition of ABC Transporter Expression and Function

Efficient adjuvants, such as ABC efflux inhibitors, are considered a promising strategy for overcoming multidrug resistance in cancer treatment. These inhibitors, developed through high-throughput screening of marketed drugs, natural products, and the design and synthesis of new modulators, have the potential to restore drug sensitivity in drug-resistant cells and alter the pharmacokinetic characteristics of anticancer drugs. Implementing these inhibitors as part of cancer chemotherapy could lead to improved therapeutic outcomes for drug-resistant cancer patients [70].

#### 3.1.1. P-gp

Gene rearrangement and tumor mutation are important mechanisms for controlling and regulating the ABCB1 gene promoter, which will lead to overexpression of the ABCB1 gene and induce drug resistance in tumors [71]. In 1981, Tsuruo et al. described the biological function of P-glycoprotein (P-gp), which is responsible for the efflux of chemotherapeutic drugs from cells. They demonstrated that the calcium channel antagonist, verapamil, can increase the sensitivity of MDR cells by functionally inhibiting P-gp. Over the following years, numerous drugs with different mechanisms emerged to restore the sensitivity of drug-resistant cells to chemotherapy drugs. Modulators of targeted P-gp activity can be categorized as follows:

(1) Direct inhibition of substrate binding sites in the P-gp domain can be achieved using competitive or non-competitive inhibitors. For instance, verapamil [19], a calcium channel blocker, act as a competitive inhibitor by competitively inhibiting P-gps binding to substrates. Conversely, HG-829 [72] functions as a non-competitive modulator of P-glycoprotein activity.
(2) Inhibiting ATP-binding domains to prevent drug translocation induced by conformational changes. For instance, GF120918 (Elacridar) [73] has been shown to effectively inhibit ATP binding and disrupt conformational alterations in P-gp, thereby impeding drug transport.
(3) Inhibiting ATP synthesis in multidrug resistance (MDR) cells through interference with glycolysis. For instance, 2-deoxy-D-glucose (2-DG) [74] has the potential to affect P-gp functionality by hindering glycolysis, thereby reducing intracellular ATP levels.
(4) Inhibition of ATP hydrolysis: oligomycin [75], an inhibitor of ATP synthase, diminishes ATP production, and, consequently, affects P-gp activity.
(5) Altering the integrity of cell membranes [76,77]. Notably, DiBenzRif [78], a derivative of rifampicin, has been shown to increase membrane fluidity, consequently inhibiting P-gp ATPase activity.

To date, three generations of P-gp inhibitors have been developed. While combinations of P-gp inhibitors and anticancer drugs have shown promising clinical results, their approval by the FDA has been hindered by their toxic side effects during clinical trials.

#### 3.1.2. MRP1

The researches on MRP1 inhibitors are still in its infancy in comparison to P-gp. Given the structural similarities between these two membrane proteins, a majority of MRP1 inhibitors also possess regulatory effects on P-gp. Notably, verapamil and cyclosporine A are recognized as typical regulators of P-gp, but they also demonstrate inhibitory capabilities on MRP1 [79]. In recent years, specific MRP1 inhibitors have been uncovered through drug design and synthesis, as exemplified by Shulinda et al. [80].

#### 3.1.3. BCRP

There are several types of BCRP inhibitors, including specific BCRP inhibitors, broad-spectrum MDR inhibitors and tyrosine kinase inhibitors [14]. The first reported specific BCRP inhibitor, Fuming arginine C, effectively reversed colon cancer resistance to mitoxantrone, but its clinical use was hindered by unexplained neurotoxicity. K0143, developed from Fumigate arginine C, not only reduces neurotoxicity, but also exhibits a 10-fold higher efficiency in inhibiting BCRP function compared to Fumigate arginine C [81]. In recent years, many ABC inhibitors have entered the clinical evaluation stage, but the results have been disappointing. The combination of these inhibitors with anticancer drugs has led to adverse effects and various degrees of toxicity, causing early termination of clinical evaluations. Despite continuous efforts in the field of multidrug resistance, no FDA-approved MDR reversal agent has been identified so far. This is primarily attributed to the toxicity resulting from the main pharmacological effects of the drugs, lack of reliable preclinical data and appropriate animal models, interference with the pharmacokinetic characteristics of chemotherapy drugs leading to cytotoxicity, and the non-specific effects of inhibitors on ABC transporters.

From a biological perspective, natural products can form associations with biological macromolecules through the process of evolutionary selection. These products possess a distinctive chemical diversity that is challenging to reproduce, thus serving as the foundation for all bioactive substances. Natural products, due to their unique structural properties, have gained recognition as promising means of discovering and developing new MDR modulators. MDR, being a complex phenomenon resulting from one or more resistance mechanisms, can potentially be overcome through the multitarget and multifunctional characteristics of natural products. As a result, some scientists advocate for the utilization of this characteristic of natural products as a more rational strategy in addressing this complex phenomenon [82].

### 3.2. Targeting Transcription Factors to Overcome Tumor Drug Resistance

Regulation of transcription factors is implicated in gene expression programming in various diseases, including cancer, neurodegenerative diseases, and diabetes. During cancer treatment, transcription factors can be activated or inhibited, resulting in the promotion or inhibition of cancer cell death. For example, the tumor suppressor protein P53 controls the expression of target genes when DNA damage occurs, which can result in stopping the cell cycle, triggering apoptosis, or causing cellular senescence [83]. Doxorubicin, a member of the anthracycline family of antineoplastic agents, functions as an inhibitor of topoisomerase II. It exerts its effect by inducing damage to DNA, thereby disrupting the processes of DNA replication and transcription [84]. This interference may lead to the emergence of MDR due to the potential loss of P53 function and overexpression of the MDR efflux pump. It has been observed that the p53 R248Q mutation in Hep3B liver cancer cells causes cross-resistance to doxorubicin and paclitaxel, possibly due to upregulation of P-gp (ABCB1) expression [85]. Furthermore, P53 regulates apoptosis-related factors BcL-2, Bcl-xL, and HO-1, which are involved in Nrf2-mediated resistance to cisplatin. This resistance can be reduced using BcL-2 inhibitors [86]. Additionally, antidiabetic drugs have been studied for their potential anticancer activity. For instance, the diabetes medication metformin disrupts MDR by modulating the ATM/LKB1/AMPK signaling pathway, proteins downstream like mTOR and MAPK, and transcription factors such as NF-κB, FOXO, and p53 [87]. Moreover, certain transcription factors such as HIF, LGK974, and FOXO play a significant role in overcoming tumor MDR [88].

### 3.3. Immunogenic Cell Death Strategy

Immunotherapy for cancer has become a vital method in combating MDR cancer. Several factors are implicated in immune therapy resistance. MDR cells exhibit robust immune avoidance capabilities due to the low expression of immune-activating molecules on their surface. Additionally, these cells can generate immunosuppressive metabolites, inhibiting the host’s immune system from mounting effective antitumor responses. The production of chemical immune resistance by MDR cancer cells is primarily driven by their ability to adapt to stressors, as part of a multi-stress resistance phenotype.

On the other hand, reactive oxygen species (ROS) have a significant role in regulating apoptosis and influencing various aspects of cancer cell behavior such as proliferation, survival, and drug resistance. Drug-resistant cancer cells generally exhibit higher levels of ROS and increased activity of scavenging/antioxidant enzymes compared to normal cells. Consequently, drug-resistant cancer cells may be more responsive to alterations in ROS levels [89]. Recently, certain small molecules have been identified as potential agents for enhancing chemotherapy sensitivity in drug-resistant cells by diminishing ROS release [90].

Developing strategies and techniques to overcome multidrug resistance is a crucial objective in current research on cancer resistance in medicine. Various chemical inhibitors and small-molecule modulators targeting the activity of transcription factors and pseudo kinases have been identified as potentially disrupting the molecular targets of multidrug resistance. However, these molecules have not yielded significant breakthroughs in clinical treatment [91]. Therefore, identifying targeted anticancer drugs that can overcome drug resistance and induce a response in resistant cancer cells remains a top priority in cancer clinical treatment. In recent years, the field of nanomedicine has gained attention for its potential to offer new approaches to MDR therapy. Nanomedicines offer advantages such as improved efficacy, enhanced bioavailability, and the ability to specifically target previously untreatable molecules, setting them apart from conventional drugs.

### 3.4. Autophagy as a Potential Strategy against MDR

Autophagy is a process through which intracellular components are degraded via endosomes. Upon cellular stress, autophagosomes are formed, encapsulating damaged organelles and proteins, and subsequently fusing with lysosomes to break down the internal material. This mechanism facilitates the removal of damaged cellular components and promotes the recycling of nutrients for cellular energy. Autophagy can potentially inhibit carcinogenesis by eliminating harmful substances and oxidative stress from cells, thus reducing cancer incidence. Conversely, cancer cells can evade drug and therapeutic stress by enhancing autophagy.

In cancer cells, autophagy plays a critical role in the context of drug resistance and chemotherapeutic stress. This occurs because chemotherapeutic drugs typically induce intracellular stress and damage, promoting the activation of autophagy, which allows cancer cells to recycle nutrients, remove damage, and sustain survival under chemotherapy-induced stress [92]. For instance, in response to stress induced by the chemotherapeutic agent cisplatin (DDP), tumor cells initiate autophagy as a survival strategy. Autophagy induces a dormant state in mitochondria within cancer cells, thereby conferring resistance to DDP. This mechanism enables the surviving cells to develop drug resistance, and in the absence of DDP, these drug-resistant cells demonstrate enhanced self-renewal capabilities and increased tumorigenicity [93].

Protective autophagy serves to shield tumor cells during the initial phases of chemotherapy treatment. Consequently, strategically modulating cellular autophagy may be an effective approach to mitigate the development of drug-resistant tumor cells. Several studies [94] have shown that inhibiting autophagy can amplify the effects of chemotherapeutic agents. For instance, 3-methyladenine (3-MA), a recognized autophagy inhibitor, effectively obstructs autophagy at the initiation and maturation stages by interfering with the interactions between class III PI3K and various ATG chaperones. The combination of 3-MA and 5-FU has been shown to enhance the therapeutic impact on gastric cancer cells. Additionally, 3-MA has been found to increase the sensitivity of paclitaxel-resistant HeLa cells to paclitaxel. These findings suggest that modulation of the autophagic pathway could be a viable strategy to overcome drug resistance.

### 3.5. Nanotechnology-Based Strategies to Overcome Multidrug Resistance

Although the discovery of multiple ABC inhibitors holds promise for the treatment of MDR, these modulators face challenges such as lack of specificity, low bioavailability, adverse side effects, drug dose-related issues, and interactions with other chemotherapeutic agents. In recent years, nanomaterials have emerged as a solution to address these challenges and provide new possibilities for the treatment of MDR tumors. Nanocarriers, including liposomes, micelles, dendrimers, and a range of organic and inorganic nanoparticles, can adjust the biodistribution, therapeutic efficacy, and toxicity of their encapsulated substances because of their unique physical, chemical, and biological characteristics. To further improve the efficacy of chemotherapy drugs, researchers have focused on surface modifications of these nanocarriers. Passive and active targeting approaches have been used to increase the accumulation of chemotherapy drugs at the target site, improve drug safety, and enable controlled drug release, thereby prolonging the circulation time of drugs in vivo and increasing bioavailability [95,96,97,98,99,100]. However, the development of nanoparticles as drug delivery carriers must address certain key issues. Firstly, they must exhibit good biocompatibility and biodegradability. Secondly, they must maintain stability under physiological conditions. Finally, they should have high drug loading capacity with low toxicity. The optimal situation would entail the precise delivery of medication directly to the desired location, thereby maximizing treatment effectiveness and reducing adverse effects. Significant progress has been made in recent years with the use of nanomaterials as carriers for targeted drugs, and some carrier systems have already been utilized in clinical practice.

The most frequently and commonly encountered nanoparticles encompass liposomes, micelles, dendritic macromolecules, quantum dots, carbon nanotubes, and metal-based nanoparticles [101,102], Table 1 provides a summary of current study seeks to elucidate the differences among various nanoparticles.

Liposomes are vesicles composed of phospholipids, capable of self-assembling into spherical shapes in aqueous environments, with diameters ranging from nanometers to micrometers. The aqueous core of these nanoparticles can be loaded with various therapeutic molecules. As drug carriers, liposomes are biocompatible and safe; they can extend drug half-lives, control release rates, and protect drugs from degradation.

Micelles are microstructures that surfactants form in aqueous solutions, consisting of a hydrophilic outer layer and a hydrophobic core with a diameter of less than 50 nm. The hydrophobic core carries hydrophobic drugs, whereas the hydrophilic layer shields the micelles from rapid degradation in physiological environments.

Dendrimer macromolecules are polymers characterized by three-dimensional hyperbranched structures that can bind a wide range of biologically active agents to form active conjugates. These agents encompass 5-fluorouracil, cisplatin, adriamycin, methotrexate, and paclitaxel.

Quantum dots (QDs) possess electronic properties beneficial for drug delivery and diagnostics, consisting of an inorganic semiconductor core and surrounded by an organic aqueous shell, typically involving a cadmium selenide core and a zinc sulfide shell. These QDs are employed as fluorescent markers within drug delivery systems and prove effective in monitoring drug biotransformation within the body.

Carbon nanotubes (CNTs), consisting of carbon atoms layers, form a cylindrical structure with diameters ranging from approximately 2.5 to 100 nm. CNTs can efficiently penetrate cells, delivering drugs directly to the cytoplasm or nucleus, and act as antioxidants, scavenging free radicals.

Metal nanoparticles (NPs), including silver, gold, copper, and zinc, can be synthesized and modified to attach to antibodies, ligands, and drugs. They are extensively utilized in biological applications owing to their high specific surface area and diminutive particle size. Nevertheless, due to the cytotoxic nature of most metals, the most stable element, gold, is frequently preferred over other metals.

#### 3.5.1. Nanostrategies for Inhibiting the Expression and Function of ABC Transporters

Small-molecule chemotherapeutic drugs predominantly traverse tumor cells via passive diffusion, but they are easily recognized and expelled by the ABC transporter proteins present on drug-resistant cell membranes. As a result, these drugs fail to effectively reach their intended target sites. Research has indicated that certain cationic polymers possess nuclear transport properties. Consequently, when drugs are enclosed within nanocarriers, they can be directed towards the cell nucleus, enhancing their efficacy. Consequently, the use of multifunctional nanoparticles that can simultaneously encapsulate antitumor drugs and MDR reversal agents has garnered increasing attention to overcome tumor MDR. Table 2 provides a summary of recent studies focusing on nanomaterials that reverse tumor multidrug resistance by targeting ABC transporters.

#### 3.5.2. Nanocarrier System with Small Interfering RNA

**Figure 3:**
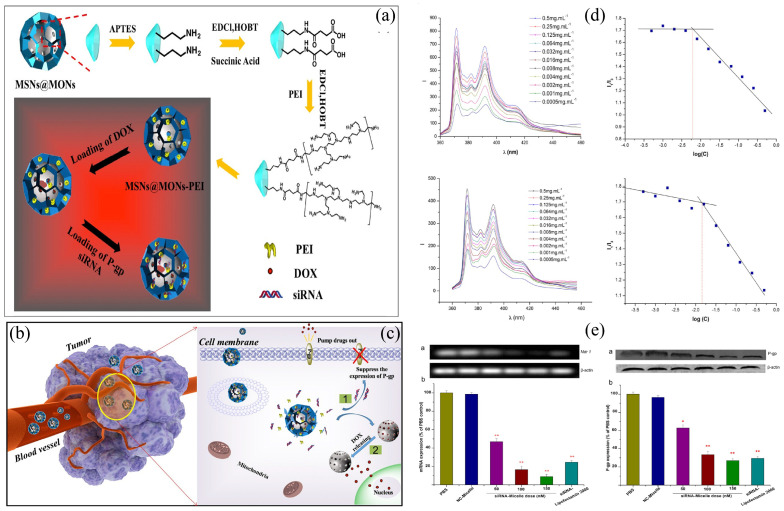
Nanocarrier system with small interfering RNA. (**a**) The mesopore surface is modified with PEI to allow for the loading of P-gp modulator siRNA. (**b**) H-MSNs co-loaded with DOX and siRNA extravasate into tumor stroma across the blood vessel and angiogenic vasculature, and finally are endocytosed into cancer cells. (**c**) Mechanism scheme demonstrating the therapeutic functions of H-MSNs in suppressing MDR of cancer cells and enhancing chemotherapy efficiency [122]. (**d**) Top: Formation of the micelleplex between NSC–PLL–PA and siRNA was determined by the quenching method using EtBr. Bottom: In vitro drug release of Dox-micelle and siRNA in different media compared with Dox [121]. (**e**) P-gp levels were detected by RT-PCR (left) or Western blot (right) [121], * *p* < 0.05 and ** *p* < 0.01 compared with the controls (n = 3).

MDR1 is the earliest discovered multidrug resistance gene, which is widely expressed in various normal cells. Studies have shown that P-gp, through its transport and secretion functions, protects normal cell tissues from the effects of cytotoxins and exogenous toxic substances. Gene silencing can interfere with the expression of specific genes in drug-resistant cell lines and has been widely used in recent years to study MDR. Small interfering RNA (siRNA), as an effector molecule of gene silencing, can downregulate the transcription of drug resistance genes, thereby restoring the sensitivity of drug-resistant cells to chemotherapeutic drugs. Zhang et al. [121]. developed a triblock copolymer micelle composed of chitosan-poly-L-lysine-palmitic acid (N-succinyl-chitosan-poly-L-lysine-palmitic acid, NSC-PLL-PA). They electrostatically adsorbed the negatively charged siRNA onto the cationic polymer PLL on the surface. The NSC component helped to avoid rapid clearance of the nanoparticles by the reticuloendothelial system, prolonging their circulation time in the bloodstream. Additionally, this micelle demonstrated pH responsiveness. Under conditions of mild acidity, the NSC-PLL-PA facilitated the expedited release of DOX and siRNA. Western blot results showed that as the siRNA concentration in the micelle increased, the intracellular mRNA level decreased, and P-gp expression was downregulated (Figure 3d,e). Sun et al. [122] also attempted to reverse MDR using a combination of siRNA delivery and chemotherapy. However, it is worth noting that large-molecule siRNA is often exposed to intracellular RNase degradation when modified on the surface of nanoparticles, resulting in decreased activity. The research team utilized a hierarchical mesoporous silica nanosystem (H-MSNs-DOX/siRNA) with large mesopores on the surface and a core structure to encapsulate the large-molecule siRNA and small-molecule DOX, respectively, thereby protecting their molecular activity. Under the action of high concentrations of glutathione in the tumor microenvironment, the disulfide bonds on the nanoparticle surface were disrupted, and the released siRNA downregulated P-gp expression, preventing drug efflux. This allowed the subsequently released chemotherapeutic drug to exert a more potent antitumor effect. Results showed that the expression of P-gp in the drug-resistant human breast cancer cell line MCF-7/ADR decreased by 40% after siRNA treatment. Confirming these findings, the subcutaneous tumor model in nude mice demonstrated that the tumor inhibition rate of the H-MSNs-DOX/siRNA group (87.0%) was higher than that of the DOX solution group (50.7%) (Figure 3). Multifunctional quantum dot nanomaterials possess the advantages of a small particle size, easy permeability, and low toxicity. Research has reported that by using electrostatic adsorption to co-load DOX and siRNA on the surface of quantum dots, the drug resistance of cervical cancer cells Hela can be reversed, and the experimental results are consistent with the research. The strategy of co-delivering siRNA and drugs can alter the efflux function of cell membrane transport proteins at the gene level, making it an effective approach to overcoming MDR, with potential significance in future studies.

#### 3.5.3. Nitric Oxide (NO) and Its Donor-Redox Responsive Drug Delivery System

**Figure 4:**
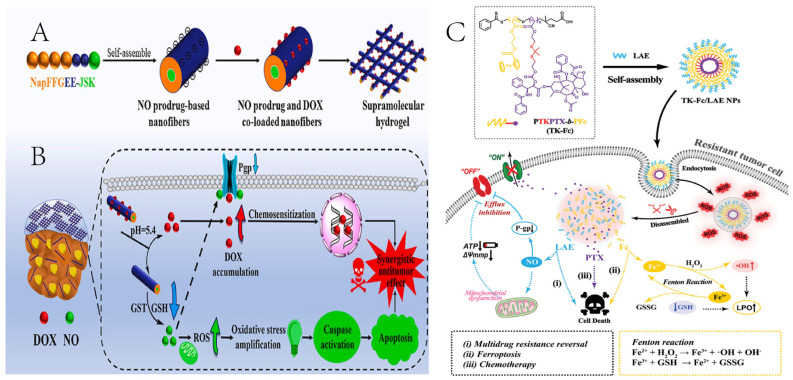
Nitric oxide (NO) and its donor-redox responsive drug delivery system. (**A**) The formation of NapFFGEE-JSK/DOX supramolecular hydrogel by self-assembly, and further loading with DOX by electrostatic and hydrophobic interactions. (**B**) The synergistic antitumor mechanism of NapFFGEE-JSK/DOX supramolecular hydrogel for combating multidrug resistance [123]. (**C**) ROS cascade nanoplatform targeting regulation of P-glycoprotein and synergistic inducing of ferroptosis to reverse multidrug resistance in prostate cancer [124].

Cancer research indicates that high concentrations of nitric oxide (NO) not only inhibit tumor growth but also have a close association with the expression of the cell membrane surface receptor P-gp. Reports propose that the inhibitory effect of adriamycin on malignant tumors might be connected to the mediation of the NO-dependent mechanism; therefore, combining chemotherapy with NO can have a sensitizing therapeutic effect. Zhang et al. successfully developed a supramolecular hydrogel using a NO prodrug through the self-assembly of the NAPFFGEE-JSK peptide. This hydrogel not only consumes a significant amount of GSH in the GSH-mediated NO release process but also generates a substantial amount of ROS by NO, thereby synergistically amplifying intracellular oxidative stress and further inducing the mitochondria-mediated apoptosis pathway (Figure 4A,B). Furthermore, this supramolecular hydrogel serves as a simple and effective codelivery platform for DOX, where the released NO can reverse the P-gp-mediated MDR effect, increasing the sensitivity of breast cancer cells to DOX, thereby achieving a synergistic antitumor effect on drug-resistant breast cancer in vivo [123]. Guan et al. reported a multimodal nanoplatform TK-Fc/LAENPs, in which the single Fc domain in the diblock copolymer first amplifies intracellular ROS levels. TK-Fc/LAE NPs are then decomposed into three parts, where TK-Fc/LAE NPs reverse MDR by generating high concentrations of nitric oxide, inhibiting NF-κB activity, and downregulating P-gp expression (Figure 4C). The NO affected mitochondrial-induced MMP, reduced ATP, and suppressed the function of the P-gp efflux pump. These processes synergistically combated MDR [124]. Chung et al. [125] previously reported that the NO donor diethylenetriamine diazeniumdiolate and camptothecin-11 were separately encapsulated in the hydrophilic and hydrophobic phases of PLGA nanoparticles for codelivery. Upon injection into the tumor, diethylenetriamine diazeniumdiolate breaks down in the acidic cellular environment of cancerous tissues, releasing a considerable amount of NO. This, in turn, destabilizes the structure of PLGA nanoparticles, enhancing the release of NO and camptothecin-11 precisely at the tumor location. The results demonstrate that PLGA nanoparticles can reduce P-gp expression by 45%, and the drug release rate of camptothecin-11 can be increased from 13% in a neutral environment to 100%. Additionally, the generated NO can serve as a contrast agent for real-time ultrasound imaging of tumor growth, thus achieving the integrated theranostic function of the drug-loaded nanoparticles. Although extensive studies have shown that NO can restore tumor sensitivity to drugs by downregulating P-gp expression, further investigation is needed to understand the underlying mechanisms and signaling pathways through which NO affects this class of transport proteins. The utilization of NO-delivering or NO donor nanoparticles to modulate tumor cell membrane transport protein function and overcome multidrug resistance may involve multiple pathways.

#### 3.5.4. Target Modification Nanostrategy to Overcome MDR

Nanocarriers, modified with targeted molecules, can enhance the accumulation efficiency of nanomedicine systems in tumor tissues, cells, or organelles by overcoming or reversing the efflux function of transport proteins. Cell-penetrating peptides (CPPs) are short peptides composed of 10 to 30 amino acid residues, typically rich in arginine or lysine. They have the ability to transport large molecular substances, such as proteins, nucleic acids, and nanoparticles, into the cell interior without causing damage to the cell structure. Pan et al. [126] employed peptide-functionalized nanoparticles to facilitate drug delivery. Through the mediation of the cell-penetrating peptide TAT, the area under the concentration–time curve of DOX in the cells increased from 463 (h·ng)/mL to 1183 (h·ng)/mL. Fluorescence analysis demonstrated that TAT nanoparticles accumulated significantly in the cell nucleus, highlighting the capability of cell-penetrating peptides to facilitate drug delivery into the nucleus, effectively circumventing efflux mechanisms mediated by cell membrane transport proteins. The study also highlighted that cell-penetrating peptides cannot directly downregulate the expression of P-gp protein. The transferrin receptor is highly expressed on the surface of acute myeloid leukemia cells. Zhu et al. [127] covalently bound transferrin to PEG-oleic acid-modified nanomaterials, forming lipid polymers. Nanoparticles can help drugs enter cells and avoid P-gp efflux. Additionally, the half-inhibitory concentration of the nanoparticle formulation (0.7 μg/mL) was 25 times lower compared to the free drug (17.6 μg/mL), indicating an enhanced cytotoxicity of the chemotherapeutic drug. Investigators have uncovered a method whereby chemotherapeutic medications, modified by affixing folic acid to the exterior of nanoliposomes loaded with the drugs, are capable of precisely navigating into tumor cells that exhibit an overexpression of folate receptors, facilitated by a mechanism called folate receptor-mediated endocytosis. Subsequently, the drugs are released from the endosomes into the cytoplasm and nucleus, avoiding the conventional delivery mode of small-molecule drugs and mitigating the influence of P-gp pumps [128]. Nonetheless, some studies have indicated that the entry of chemotherapeutic drugs, loaded onto nanomaterials, into cells may be facilitated by the interaction between cells and nanoparticles, rather than through endocytosis.

#### 3.5.5. External Responsiveness of Multifunctional Nanoparticles to Overcome MDR

The external responsiveness of multifunctional nanoparticles refers to their ability to regulate drug release, aggregation, or chemical reactions using external stimuli such as magnetic fields, lasers, and ultrasound. This capability can enhance therapeutic efficacy while reducing toxic side effects. The combination of externally responsive nanoparticles and chemotherapy shows great potential in overcoming multidrug resistance (MDR). Photothermal therapy is a promising and minimally invasive cancer treatment method. By utilizing near-infrared (NIR) light-absorbing materials, NIR laser irradiation can convert NIR into thermal energy, effectively killing tumor cells. This approach offers advantages such as deep tissue penetration and high safety [129]. The efficacy of photothermal therapy relies on the photothermal conversion capability of the materials used. The nanomaterials currently employed in photothermal therapy include noble metal nanomaterials, carbon nanomaterials, transition metal sulfides or oxides, and organic small-molecule drugs. Photothermal therapy can be combined with chemotherapy or radiotherapy to enhance the sensitivity of tumor cells to the respective treatments, thereby improving therapeutic outcomes. This synergistic therapy has shown promising results in overcoming multidrug resistance in tumors. Indocyanine green (ICG) exhibits excellent near-infrared properties and biocompatibility, and it is approved for clinical use by the US FDA. ICG demonstrates strong absorption in the near-infrared region, facilitating near-infrared imaging through near-infrared fluorescence. Moreover, it can generate free radicals and singlet oxygen for photodynamic therapy, as well as heat for photothermal therapy [130,131]. Zheng et al. developed DOX and ICG-loaded PLGA-lecithin-PEG nanoparticles via a one-step ultrasound method. Their combined chemotherapy and photothermal therapy strategy effectively inhibited MCF-7 and MCF-7/ADR tumors in vitro and in vivo [132]. Dong et al. [133] utilized the fine particle size and high photothermal conversion efficiency of molybdenum disulfide (MoS2) to design a hyaluronic acid (HA)-modified MoS2 nanosheet, aiming to reverse MDR through a combination of photothermal and chemotherapy. Under 808 nm laser irradiation, the temperature of MoS2 increased to 52 °C within 10 min, effectively killing tumor cells and significantly downregulating the expression of cell membrane transport proteins. Fluorescence imaging showed that the fluorescence distribution time of DOX was 2.2, 3.3, and 4.6 ns in the free DOX, MoS2, and MoS2-HA groups, respectively. Moreover, a significant fluorescence signal was still observed in the MoS2-HA group after 12 h of incubation with cells. In vivo experiments demonstrated that the tumor growth inhibition rate of the MoS2-HA + laser group reached 96%, which was statistically significant compared to the other groups. These findings indicate that MoS2-HA/DOX effectively delivers drugs and maintains intracellular drug concentration, thus enhancing the efficacy of photothermal–chemotherapy combination therapy.

Wang et al. [134] successfully created a lipid-membrane-coated carbon–silicon hybrid nanoparticle for delivering the chemotherapeutic drug DOX. This nanoparticle system is capable of targeting mitochondria and generating reactive oxygen species under 780 nm laser irradiation. As a result, intracellular ATP is consumed, and the ATP-dependent efflux function of the P-gp protein is temporarily impaired for at least 5 days. This leads to the establishment of a therapeutic window for chemotherapy. The effectiveness of this nanoparticle was further confirmed in drug-resistant cell models for paclitaxel and irinotecan, highlighting its potential to overcome broad-spectrum tumor drug resistance. In summary, photothermal therapy shows great promise as an approach for cancer treatment. Magnetic nanoparticles possess dual functionalities, acting as both drug delivery carriers and magnetic resonance imaging agents. Elumalai et al. [135] addressed the issue of transport protein efflux by designing multilayer magnetic nanocapsules. In their study, a 2.0 T magnetic field and a 1 cm × 1 cm bar magnet were positioned beneath the cell culture dish to facilitate the continuous accumulation of nanocapsules within the region of the external magnetic field. Consequently, the application of the external magnetic field effectively reduced the drug efflux mediated by P-gp, leading to a significant increase (87.78%) in the apoptosis rate of drug-resistant cells. Cisplatin-based combination chemotherapy is a commonly used treatment for non-small-cell lung cancer. However, studies have demonstrated that the upregulation of lung resistance protein (LRP) in the drug-resistant lung cancer cell line A549/DPP can confer insensitivity to chemotherapy [136]. To address this issue, researchers synthesized cisplatin-loaded magnetic iron oxide nanoparticles. Co-incubation of these nanoparticles with A549 cells for 48 h resulted in a significant decrease in LRP activity and a reduction in phosphorylated protein kinase Akt activity. Moreover, the tumor inhibition rate was higher compared to both the free cisplatin group and the control group, indicating the potential of this approach to overcome multidrug resistance [137,138]. Thus, the study suggests that the elevated expression of LRP, coupled with the Akt pathway, may contribute to the development of multidrug resistance.

#### 3.5.6. Additional Nanostrategies for Overcoming Tumor MDR

**Figure 5:**
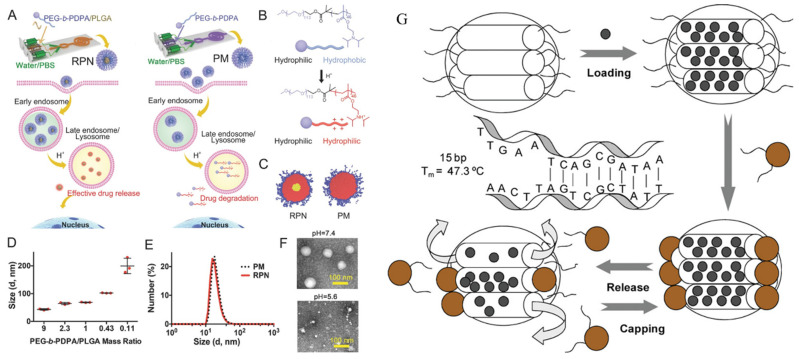
Chip-based fabrication of rigid pH-sensitive micellar nanocomplex (RPN). (**A**) Schematic of the microfluidic chip and illustration of intracellular translocation of RPN and PM. (**B**) Chemical structure of diblock copolymer (PEG-b-PDPA) which could be activated in acidic endo-/lysosomes. (**C**) Simulations to show the structure of RPN and PM. (**D**) Hydrodynamic sizes of RPN with different mass ratios of PEG-b-PDPA/PLGA measured by DLS. (**E**) Hydrodynamic sizes of PM and RPN with the mass ratio of PEG-b-PDPA/PLGA of 9. (**F**) TEM images of RPN at pH 7.4, and dissociated RPN at pH 5.6 [139]. (**G**) Reversible magnetic nanogates drive drug release from magnetic mesoporous silica particles through DNA hybridization/dehybridization [141].

In addition to the methods mentioned above, drug resistance related to cell membrane transport proteins can also be overcome with multifunctional nanoparticles that are designed and constructed based on rational design. Once internalized into the cell, these nanoparticles typically reach the lysosome. Effective action of the nanoparticles is only possible when they can successfully escape from the lysosome. Certain cationic polymers, such as polyethyleneimine and polyamidoamine dendrimer, exhibit a “proton sponge” effect. When these cationic polymers enter the acidic environment of the lysosome, their unprotonated amino groups capture a significant number of protons. This process leads to the accumulation of chloride ions and water molecules in the lysosome, causing it to swell and eventually rupture. As a result, the contents of the lysosome are released, allowing the cationic polymers to escape successfully. Feng et al. [139] synthesized a double-hydrophilic block copolymer, poly (ethylene glycol)-b-poly(2-(diisopropylamino)ethyl methacrylate) (PEG-b-PDPA), using a one-step method. By utilizing the pH-responsive protonation of PDPA, the nanoparticles can achieve “lysosomal escape” in the weakly acidic tumor microenvironment. This not only reduces the efflux of anticancer drugs by cell membrane transport proteins but also decreases the impact of lysosomal hydrolases on drug activity. The study also suggests that incorporating PLGA into the nanoparticles can increase the material’s hardness, improving the efficiency of cellular uptake. The results reveal that, when using this nanomaterial to deliver DOX, the drug efflux rate was reduced from 80% in the DOX-only group to less than 20%. The efficient accumulation of the drug helps to reverse multidrug resistance and exert the antitumor effect (Figure 5A–F). Furthermore, reports indicate that the addition of surface-modified active agents to polymers can disrupt the lipophilic environment of the cell membrane, causing changes in the secondary and tertiary structures of cell membrane transport proteins and effectively reversing tumor multidrug resistance. However, this method has not yet been confirmed by in vivo experiments. Kim et al. evaluated the feasibility of an exosome-based drug delivery system for the treatment of drug-resistant tumors [140]. Exosomes derived from macrophages loaded with paclitaxel (PTX) were subsequently reconstituted through sonication to enhance drug encapsulation efficiency. Within the drug-resistant MDCK-MDR1 cells, the PTX-encapsulated exosomes exhibited a 50-fold enhancement in antitumor efficacy in comparison to unencapsulated PTX. There have been reports of connecting a single-stranded DNA with iron oxide nanoparticles, where the two DNA strands form a double helix structure, encapsulating the drug within the mesoporous silica interior [141]. Under normal conditions, there is almost no drug release from these nanoparticles. However, upon application of an alternating magnetic field, the magnetic nanoparticles emit heat, triggering the disentanglement of DNA and the liberation of the drug imprisoned within the mesoporous silica, thereby achieving precise control over drug release (Figure 5G).

### 3.6. Joint Strategy against MDR

The term “combination therapy for tumor resistance” refers to the concurrent administration of multiple therapeutic agents, including drugs, targeted therapies, and immunotherapies, aimed at circumventing tumor cell resistance to singular treatments. The advantages of this approach include: (1) The utilization of diverse pharmacological agents and modalities to target different tumor mechanisms, thus enhancing overall efficacy; (2) the reduced likelihood of tumor cells developing drug resistance; (3) by combining multiple strategies, the treatment success rate and survival rate can usually be improved; and (4) tailored combination regimens according to specific patient conditions for more precise therapeutic effects.

A researcher [142] has developed a functionalized polymer nanoparticle from poly(γ-glutamic acid)-g-poly(lactic acid-hydroxyacetic acid copolymer), loaded with doxorubicin (DOX) and indocyanine green (ICG), for dual-modality cancer therapy. These nanoparticles were coated with cholesterol-PEG (C-PEG), allowing for photothermal-triggered intracellular drug release. Post light irradiation, the C-PEG-coated DI-NPs exhibited a synergistic chemotherapy and thermotherapy effect, effectively inhibiting the proliferation of MDR cancer cells.

Immune checkpoint inhibitors (ICIs) represent a class of pharmaceutical agents [143] utilized in cancer treatment. These medications bolster the immune system’s capacity to recognize and eradicate cancerous cells by inhibiting immune checkpoint proteins. Incorporating combination therapies can amplify the potency of ICIs, mitigate side effects, and avert drug resistance. For illustration, coupling RAF/MEK inhibitors with ICIs has led to notable improvements in treatment outcomes and lowered recurrence rates among patients harboring the BRAF V600E mutation. Additionally, the combination of BRAF and MEK inhibitors with ICIs [144], including darafenib, trametinib, and spadalizumab, has elicited a favorable response in this patient population. As an additional example, the combination of HER-2-targeted therapy with ICIs demonstrated favorable outcomes in HER2-positive breast cancer patients [145]. The combination of trastuzumab and pembrolizumab [146] exhibited promising results, including enhanced response rates and prolonged progression-free survival in PD-L1-positive tumors. Furthermore, the integration of artificial intelligence and machine learning techniques may improve immunotherapy by predicting optimal synergistic drug combinations for cancer treatment [147].

The employment of bioactive natural compounds alongside conventional chemotherapeutic agents has proven effective in treating various cancers, including breast, prostate, glioblastoma, and colorectal cancers. Integrating standard chemotherapeutic drugs with bioactive natural compounds is a promising approach for counteracting cancer drug resistance. Natural bioactive products derived from plants have shown to enhance drug efficacy at lower doses, mitigate dose-related toxicity, and re-sensitize drug-resistant cells via multiple molecular mechanisms. For instance [148], curcumin, resveratrol, and EGCG are among the most extensively studied plant-derived compounds that potentially augment the effectiveness of conventional chemotherapeutic drugs and counteract drug resistance. The concomitant treatment with resveratrol and adriamycin led to the upregulation of the pro-apoptotic gene Bax in the colorectal HCT116 cell line [149], thereby enhancing apoptosis. Concurrently, the treatment limited drug efflux through the downregulation of ABCB1, which increased the intracellular concentration of the drug and its therapeutic effect. It has been shown that combining traditional chemotherapy drugs with curcumin is an effective approach to overcoming drug resistance [150]. It was observed that the combination of EGCG and cisplatin led to an enhancement of autophagy in DLD-1 and HT-29 colorectal cancer cells, which was induced by cisplatin [151]. The evidence was seen in the buildup of LC3-II protein, a greater increase in acidic vesicular organelles (AVOs), and the creation of autophagosomes.

## 4. Conclusions and Future Perspectives

The formation of multidrug resistance (MDR) is influenced by various factors. Among these factors, the efflux of chemotherapeutic drugs mediated by ABC transporters is a prominent area of research in MDR studies. In recent years, the study of drug resistance-related proteins has led to promising results in reversing MDR by targeting cell membrane transport proteins. However, solely inhibiting these proteins is insufficient to achieve the desired outcomes. The use of nanocarriers to deliver drugs in antitumor therapy has numerous advantages compared to traditional drug delivery methods. It circumvents the constraints of conventional drug delivery and enhances drug efficacy by integrating various methods to counteract MDR. Nano-formulations can reach the tumor site through passive or active targeting, leading to increased drug concentrations at the tumor site. These formulations primarily enter cells via endocytosis, shielding the drugs from recognition and expulsion by efflux proteins, thereby increasing intracellular drug concentrations. Nano-delivery systems can also be engineered to precisely target specific organelles, allowing for stepwise targeting and the effective delivery of drugs to the desired sites. Moreover, nano-delivery systems enable the delivery of various types of drugs, such as chemotherapeutic drugs, MDR reversal agents, gene drugs, and photosensitive drugs, either alone or in combination. Therefore, nano-formulations offer multiple strategies for overcoming tumor multidrug resistance.

However, there are several issues that arise in practical applications. Firstly, while the development of MDR reversal agents has made significant progress, it is important to note that P-gp and other related transport proteins are not only found in tumor cells but also widely distributed in normal cells. Hence, striking a balance between inhibiting the efflux of antitumor drugs and maintaining the body’s homeostasis is crucial for the effective utilization of MDR reversal agents. Secondly, drug delivery processes often encounter challenges such as the instability of sensitizing agents and the difficulties associated with long-term storage in nano-systems. Therefore, further research is needed to identify ways to effectively preserve the drug’s activity within nanoparticles and successfully release it in the target area. Thirdly, it is important to highlight that a majority of existing studies on this subject remain at the in vitro experimental stage. Therefore, extensive in vivo experiments, as well as phase II and III clinical trials, are necessary to validate the effectiveness and reproducibility of these findings. It is anticipated that the comprehensive development of multifunctional nano-platforms will offer new opportunities to address the aforementioned issues in the future.

## Figures and Tables

**Table 1:** Current study seeks to elucidate the differences among various nanoparticles [103,104,105,106,107].

Material Type	Definition	Characterization	Example
Solid nanoparticles	Particles with dimensions between 1 and 1000 nanometers that typically exhibit a high specific surface area	Nanoparticles comprised of a single material or a composite with tunable surface properties that are suitable for drug encapsulation and release	Polymer nanoparticles, exemplified by PLGA, as well as silica nanoparticles and solid lipid nanoparticles (SLNs), are of particular interest
Hydrogel	The material is composed of polymers with a three-dimensional network structure, which enables it to absorb significant quantities of water and form gels with a high moisture content	The material exhibits a high hydration capacity, flexibility, and good biocompatibility, which are conducive to controlled drug release	Polyvinyl alcohol (PVA) hydrogel, polyacrylic acid (PAA) hydrogel
Metal nanoparticles	Metal particles are defined as particles between 1 and 1000 nanometers in size, which are typically composed of a single metal or metal alloy	They possess distinctive optical, electronic, and catalytic characteristics, including the surface plasmon resonance effect and antimicrobial properties	Gold nanoparticles (AuNPs) and silver nanoparticles (AgNPs)
Nanofibers	Fibrous materials with diameters on the nanoscale (typically in the range of tens to hundreds of nanometers) and lengths that can reach microns or longer	The high specific surface area, good mechanical strength, and high porosity of these materials are utilized in a variety of applications, including drug delivery and tissue engineering	Nanofibers composed of polylactic acid (PLA) and polyurethane (PU) are also utilized in this field

**Table 2:** Recent reports on nanostrategies for inhibiting the ABC transporters.

Carrier System	Drug	Composition	Preparation Method	Tumor Cell Lines	MDR Reversal Mechanism	Vitro/Vivo	Reference
Liposome	DOX	Dimethyldidodecylammonium bromide, 1,2-dipalmitoyl-sn-glycero-3-phosphocholine	Chemical synthesis	DOX-resistant MCF-7 and HL-60 cell lines	Evade P-gp function.	Vitro	[108]
DOX- assembled nanoparticles	DOX	DHA, Tween 80, octadecenic acid, DOX, docosanoic acid, soybean phospholipids, DSPE-PEG2000	High-pressure microfluidic technique	DOX-resistant HepG2 cell lines	DOX-nano drug delivery system decreases the expression of drug efflux-related proteins.	Both	[109]
Silver nanoparticle	CPT	Silver nitrate	Antisolvent precipitation method under ultrasonic illumination.	A549, MCF-7 HeLa, MDAMB231, SKBR3	P-gp expression and activity inhibited.	Vitro	[110]
EVs-based nanocarriers	DOX	Lemon EVs, succinylated heparin, cRGD peptide, 1-ethyl-3-(3-dimethylaminopropyl) carbodiimide hydrochloride, N-hydroxysuccinimide et al.	Chemical synthesis	DOX-resistant ovarian cancer cells	HRED can reduce adenosine triphosphate; drug efflux has significantly reduced.	Both	[111]
Solid lipid nanoparticles	DTX-PL	Phospholipid, cetyl palmitate/stearic acid/glycerol monostearate and tween 80	Microemulsification technique	MCF-7 cancer cell lines	SLN reduces the drug exposure to the ABC efflux pump.	Both	[112]
Carbon-based nanocarriers	DOX	N-hydroxysuccinimide, sulfuric acid, nitric acid, 1-Ethyl-3-(3-dimethylaminopropyl) carbodiimide, carbon nanotubes	Chemical synthesis	H69AR, DOX drug-resistant tumor cell lines	CNT-DOX can induce cellular accumulation in tumor cells, inhibiting ATP by mitochondrial damage.	Both	[113]
Solid lipid nanoparticles	Taxol	Oleic acid, glycerol monostearate, lecithin, TPGS, Brij 78	Emulsification evaporation–low temperature solidification method	Paclitaxel-resistant human ovarian carcinoma cell lines	The MDR tumor can be sensitized by Cur-SLN through the inhibition of the P-gp drug efflux pump.	Vitro	[114]
Peptide nanofiber hydrogel	DOX	RADA16-R8 powder, RADA16 powder, NaCl solution	Chemical synthesis	Multidrug-resistant SKOV3 cell line	RRD hydrogel increases the absorption and penetration of drugs into cancer cells by enhancing various internalization pathways and reduces drug efflux by inhibiting the P-gp and BCRP multidrug resistance transporter proteins.	Both	[115]
Self- assembled nanoparticles	DOX	mPEG, peptide, DOXO-EMCH	Chemical synthesis	Multidrug-resistant MCF-7 cell lines	MSNPs can overcome sequential physiologyical barriers of multidrug resistance by passing ABC transporter efflux by undergoing receptor-mediated endocytosis.	Both	[116]
Gold nanoparticles	Bilirubin	Auricchloride, sodium citrate. Conjugated with folate	Chemical synthesis	P-gp overexpresses KB-ChR-8-5 cell lines	Bypasses the MDR in cancer cell line.	Both	[117]
Solid lipid nanoparticles	Taxol, GRh2	Glyceryl monostearate, cremophor EL, beta-estradiol 3-benzoate, coumarin 6, 20(s)-Ginsenoside Rh2(GRh2)	Modified melt-emulsion ultrasonication and low-temperature solidification approach	Paclitaxel-resistant MCF-7 cell lines	SLNs-Gel reduce the expression of P-gp and the content of adenosine triphosphate in cells; make PTX-resistant breast cancer cells (MCF-7/PTX) sensitive to PTX.	Both	[118]
Self-assembly-enabled	Ferrostatin-1 Dox	Ferric chloride hexahydrate, 1,4-benzenedicarboxylic acid, pseudolaric acid B	Chemical synthesis	DOX-resistant MCF-7 cell lines	Regulation of cell membrane fluidity and permeability.	Both	[119]
Silver nanoparticles	Ag20	AgNPs	None	A549, HepG2 (HB-806), SW620 (CCL-22) cell lines	Ag20 has a toxic effect on the test cells and regulates the expression and transport activity of ABC proteins.	Vitro	[120]
